# Nonresonant 10^4^ Terahertz Field Enhancement with 5-nm Slits

**DOI:** 10.1038/srep45638

**Published:** 2017-04-03

**Authors:** Om Krishna Suwal, Jiyeah Rhie, Nayeon Kim, Dai-Sik Kim

**Affiliations:** 1 Department of Physics and Astronomy and Center for Atom Scale Electromagnetism, Seoul National University, Seoul 08826, Republic of Korea

## Abstract

Transmission of Terahertz (THz) electromagnetic wave through a substrate is encumbered because of scattering, multiple reflections, absorption, and Fabry–Perot effects when the wave interacts with the substrate. We present the experimental realization of nonresonant electromagnetic field enhancement by a factor of almost 10^4^ in substrate-free 5-nm gold nanoslits. Our nanoslits yielded greater than 90% normalized electric field transmission in the low-frequency THz region; the slit width was 5 nm, and the gap coverage ratio was 10^−4^ of the entire membrane, 0.42 mm^2^. This large field enhancement was attributed to gap plasmons generated by the THz wave, which squeezes the charge cross-section, thus enabling very highly dense oscillating charges and strong THz field transmission from the nanoslits.

Field enhancement has received widespread attention since the first report, in 1998, of extraordinary optical transmission (EOT) from a subwavelength aperture in a metal based on surface plasmon polaritons (SPP)^1^, because of its many potential applications, including nonlinear plasmonics^2^,^3^, optical antennas^4^,^5^, high harmonic electromagnetic wave generation^6^,^7^, optical rectification^8^, surface enhanced Raman scattering^9^ and metamaterial sensors^10^,^11^,^12^, nonlinear optics^2^,^13^,^14^, and superlenses and cloaking^15^. Field enhancement factors of several orders of magnitude have been demonstrated with various geometric configurations, such as metallic gaps, slits, apertures, metal particles, and tips^16^,^17^,^18^,^19^,^20^,^21^,^22^. In these configurations, the dominant phenomenon is the distribution of oscillating charge carriers (local plasmons) around the gap, which is generated by the electromagnetic wave at the interface between the metal and dielectric layers, thus creating strong electric and magnetic fields around the interface and enhancing optical phenomena. For convenience, the enhancement factor is generally defined as the electric field enhancement factor, F(ν), which is given as F(ν) = E_gap_(ν)/E_inc_(ν), where E_gap_(ν) and E_inc_(ν) represent the electric field at the gap and incident point, respectively, for the given frequency, ν.

In THz field transmission, field enhancements of several orders of magnitude have been obtained with a nanometre-wide slit^22^,^23^,^24^. Previously, we have demonstrated enhancement factors of nearly three orders of magnitude with a 70-nm-wide slit^21^. M. Shalaby *et al*. have also demonstrated nearly a three-orders-of-magnitude THz field enhancement by using an optimized periodic array of −40 nm slits^22^. Recently, Y.M. Bahk *et al*.^25^ have demonstrated an approximately 7000-fold electric field enhancement by using a copper-single layer graphene-copper (C-G-C) hybrid structure. A prominent question is how large a THz field can be enhanced by using nanoslits. We assumed that a major obstacle is the substrate, which decreases the transmission and creates undesirable interactions between the gap and the substrate. Moreover, multiple reflections (Fabry–Perot resonances) from the substrate degrade the signal quality. However, to generate fully free-standing nanoslits, substantial fabrication challenges must be overcome to allow tolerance of internal and external stresses from the membrane itself.

Here, we demonstrate the experimental realization of very large field enhancement by using gold nanoslits on a Si_3_N_4_ membrane. Our methods were based on atomic layer lithography combined with multiple layers of metal deposition and selective etching^26^. Here, the slit width was determined by using the atomic layer deposition (ALD) thickness in an optically opaque metal membrane. Our nanoslits had a 5-nm gap that extended up to the entire membrane, with a length of 0.65 mm that was sufficient to surpass the cut-off THz frequency. This structure enabled us to achieve near-maximum field enhancement in the THz region.

## Results and Discussion

**Figure 1:**
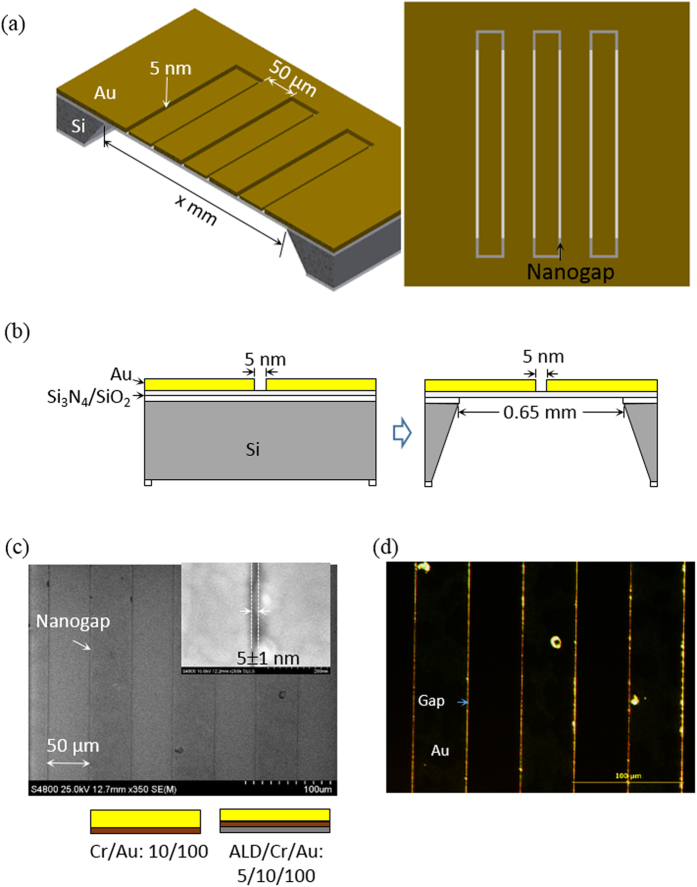
Nanoslit sample description. (**a**) Schematic drawing and the top view of a half section; (**b**) cross-section showing the process to make substrate-free nanoslits. (**c**) SEM image showing the nanoslit array from the back. The inset shows the nanogap in the metallic film. (**d**) Optical micrograph of the nanoslit array when the plane of polarization is perpendicular to the slit.

For the experimental realization of the very large field enhancement by using subwavelength nanoslits, we fabricated 5-nm slits in 100-nm thick gold film on a 1-μm SiN_x_ membrane and then performed THz time domain spectroscopy (TDS) over the frequency range 0.05–1.7 THz (wavelength 6–0.175 mm) in transmission mode. Our nanoslits had nanometre-wide metallic slits that extended along the entire length of the membrane, thus maintaining the uniform air gap throughout the length, as shown in the half-section schematic and top view presented in Fig. 1a. Nanoslits with gold film on the SiN_x_ membrane were supported by the robust Si frame.

We first fabricated the nanoslits on the SiN_x_-coated Si substrate. Then, bulk Si was etched out through the back-side window, as presented in the schematic cross-sectional diagram in Fig. 1b. The fabrication details will be described in the methods section. Figure 1c shows a scanning electron microscopy (SEM) image of the top view of the nanoslits. The inset of Fig. 1c depicts the gap formation in the metal film, which was measured to be 5 ± 1 nm. In the optical image (Fig. 1d), light scattered through the 5-nm gap was clearly observed. The slit was not visible when the polarization was turned to 90°. The optical transmission of white light through the nanogap indicated the clear formation of the nanogap, and the diffracted light of the longer wavelength was transmitted, whereas the transmission was null when the light was polarized along the slit direction.

**Figure 2:**
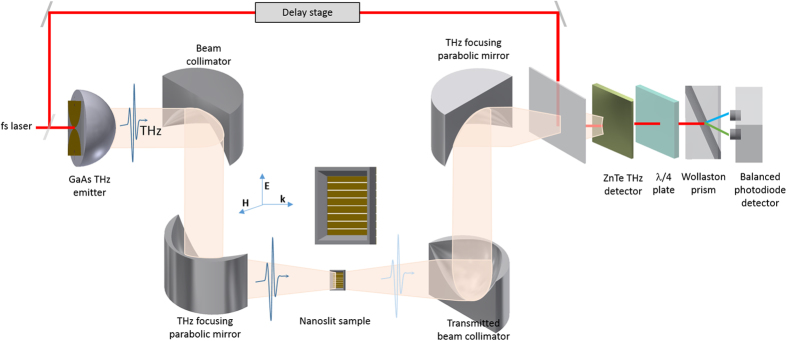
Schematic of THz time domain spectroscopy (TDS) measurement. Femtosecond laser pulses were divided for THz generation and detection. Broadband THz generated by the biased GaAs emitter was plane polarized, collimated and then focused on the nanoslits, and the transmitted beam was detected with a ZnTe crystal. A Wollaston prism separated the beam into two orthogonal, linearly polarized beams for the electric field amplitude measurement using a balanced photodiode detector. The TDS was obtained for both the TM and TE modes after the TE mode was subtracted from the TM mode to obtain the THz field amplitude through the nanoslit.

Figure 2 presents a schematic diagram of the THz-TDS measurement setup. For this experiment, a mode-locked Ti: sapphire femtosecond laser source (central wavelength: 780 nm, pulse width: 130 s, and frequency: 80 MHz) was split to generate and detect THz radiation. One of the beams impinged on the biased GaAs semiconductor crystal and emitted broadband THz from the metallic antenna patterned on the crystal. Then, THz was collected by a Si hemisphere and plane polarized before it was focused on the sample. The p-polarized THz pulses were normally focused on the nanogap slit. The transmitted waves were collected using parabolic mirrors (NA = 0.32) and detected via electro-optic sampling using a (110)-oriented ZnTe crystal. The THz time domain spectra were mathematically transformed into frequency domain spectra (FDS) via Fourier transformation. A Wollaston prism split the x- and y-polarized beam by using a balanced photodiode detector for the electric field amplitude measurement. The TDS was obtained for both the transverse magnetic field (TM) and transverse electric field (TE) modes. Later, the TE mode was subtracted from the TM mode to obtain the THz field amplitude through the nanoslit. The measurement was performed in both TM and TE modes; later, the enhancement of the normalized field amplitude was calculated by subtracting the transmission signal in the TE mode from that in the TM mode. The TM and TE modes were measured by focusing the plane-polarized THz beam on the nanoslit sample at 0° and 90° by simply rotating the sample by 90°.

**Figure 3:**
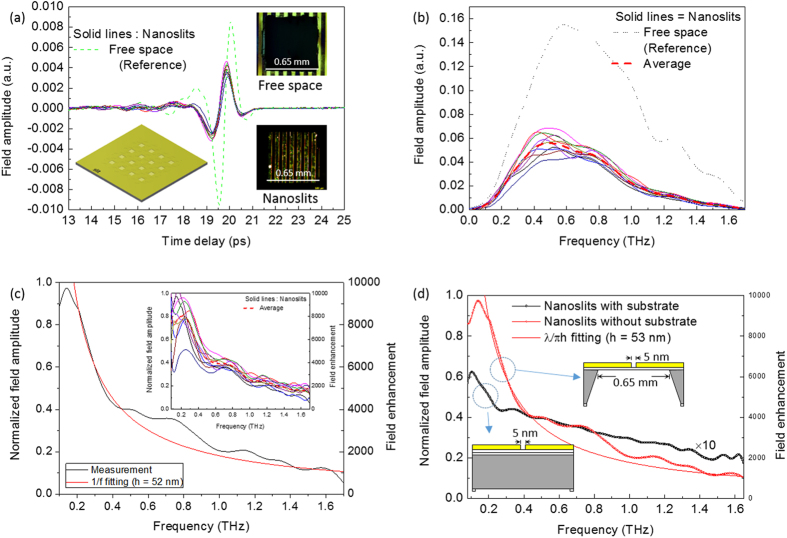
THz transmission measurements through the gold nanoslits. (**a**) TDS of the gold nanoslits and free space transmission from the aperture area 0.65 × 0.65 mm^2^. Inset: optical micrographs of reference and nanoslit membrane samples and schematic of nanoslit membrane array. (**b**) FDS spectra obtained by computing the Fourier transformation of the TDS. (**c**) Field amplitude normalized to free space transmission and field enhancement factor with 1/f fitting. (**d**) Field amplitude before and after the substrate was etched out.

Figure 3a shows TDS data obtained from the gold nanoslits and the free space transmission from the aperture area, 0.65 × 0.65 mm^2^. The peak transmission amplitude through an array of 5-nm slits, which occupied only 0.01% of the membrane area, was as high as 50%. In contrast, the direct THz transmission in the TE mode was less than 0.1% (not shown), thus indicating very strong field enhancement inside the slits. The transmitted THz time trace data demonstrated that the periods of fundamental oscillation passed through the nanoslits shifts 150 fs earlier than through the free space aperture. The shift indicated significant changes in the effective refractive indices. A typical reference and nanoslit sample used to measure TDS is presented on the right side of Fig. 3a (upper and lower optical micrographs). The lower left panel shows a schematic of a sample with 4 × 4 nanoslit membrane arrays in a 10 × 10 mm^2^ Si substrate.

We obtained FDS, as shown in Fig. 3b, after generating the spectra by Fourier transformation of the TDS. Figure 3b shows the field amplitude versus the frequency of the THz transmission from the nanoslits (solid curve), which exhibits a similar trend as the free space (dotted curve), thus indicating non-resonance transmission. The red dashed curve indicates the average field amplitude of the transmission of 10 samples. Furthermore, the field amplitudes were normalized to the free space transmission and are presented in Fig. 3c. The graph shows that the normalized field amplitude decreased with increasing frequency without any resonance. The experimental data were directly fit to 1/f, which is represented by the red curve in the graph, where f is the frequency. The non-resonance behaviour was attributed to the very high length-to-width ratio (1.3 × 10^5^) of our nanoslits, which caused them to behave as infinite nanoslits.

To calculate the field enhancement factor, we used diffraction theory^27^ because our nanoslits were made of optically opaque gold, which has a dielectric constant that is ~106 times that of air in the THz range. We measured the far-field THz-TDS from free space and from the nanoslit sample with equal-sized apertures. The transmitted electric field from free space and from the nanoslit sample were 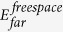 and 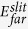; then, the normalized amplitude, t(ν), was defined as the ratio of these two measured amplitudes: 
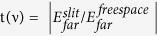
. Division t(ν) by the aperture coverage, β, yields the field enhancement factor, F(ν), in the nanogap region: F(ν) = t(ν)β, where β = A_slit_/A_freespace_ = w/p (for slit width, w, and period, p).

We observed a straight 1/f-dependent field enhancement factor, as shown in Fig. 3c, where f is the frequency. We used the fitting formula λ/πh, where λ is the wavelength, and h is the nanogap depth. The curve was fit to the data at h = 52 nm, which represents the effective thickness of the gold nanogap. The field enhancement was approximately twofold larger than the value given by λ/πh when we used the actual film thickness in the calculation. This observation indicated that the effective thickness of the metal film was almost half that of the actual thickness, in agreement with an asymmetric shape with only half of the metal thickness forming the 5-nm gap. This result agreed with findings previously reported by S. Han *et al*.^17^ The plot demonstrates that the field enhancement decreases with increasing frequency, almost monotonously, with a 1/f dependency. Higher field enhancement was observed at the lower frequency, which was almost ten thousand at approximately 0.1 THz. In our setup, the minimum reliable frequency with which we could observe the drop in the field enhancement was 0.05 THz. We believe that the enhancement was saturated beyond this frequency. Thus, 1/f fitting was valid for the enhancement factor beyond the peak. The normalization was completed with the free space transmission through an aperture that was made with optically thick aluminium foil of the exact same size as the membrane. The experimental results of the 10 different samples are shown in the inset.

To explain the very high field enhancement through the nanoslit without the substrate, we simply assumed that the nanoslit was a nanocapacitor that enabled the storage of a large charge. When THz waves pass through the nanoslits, the sidewalls of the slits act as nanocapacitors, owing to the oscillating opposite charge carrier concentrated around them. The charge density is proportional to the incident field and inversely proportional to the gap width. For narrow gaps, the charge density creates a very strong electric field that enhances the signal by several orders of magnitude relative to the incident electric field. The charges at the edges of the metallic slit oscillate with the alternation of the incident electric field and re-radiate the electromagnetic wave, where the gap acts as an antenna. The re-radiated power depends on the charge density or the electric field at the gap. By measuring the re-radiated electromagnetic waves, we estimated the field enhancement factor at the slit. The first experimental realization of a nonresonant field enhancement factor of nearly 1,000 was previously demonstrated with a 70-nm-wide gold slit^21^. Our current measurement with 5-nm-wide nearly free-standing nanoslits is presented in Fig. 3c; the enhancement was nearly 10,000 at the lower frequency near 0.1 THz. Because both results demonstrated the same 1/f dependence, the decrease in the gap width markedly increased the enhancement factor, up to nearly 10^4^, at the frequency 0.14 THz. Although our field enhancement factor was less than half of the resonant transmission through a slot antenna array with a nearly 1-nm gap, as reported by X. Chen *et al*.^28^, it is the highest nonresonant enhancement factor ever reported in the scientific literature. Moreover, our nanoslit array was nearly transparent to the THz field, even though the metal thickness was half of the skin depth at that frequency, whereas the aforementioned work^28^ reported only 60% transmission from the 5-nm gap. It is important to note that the skin-depth of gold at this frequency was 201 nm, which was 40 times that of our slit width and double the thickness of our gold film, thus indicating that electromagnetic waves were not only highly squeezed at the nanogap but also highly re-emitted through the nanogap. In our case, the gap width was a million times smaller than the wavelength; however, the length of the slit was of the same order as the wavelength.

In order to analyse the substrate effects in transmission and the field enhancement factor, we performed TDS measurements with nanoslits on substrate and without substrate. Our substrate was bulk silicon (Si 〈100〉, thickness: 300 μm, resistivity: 10 ohm.cm), and we obtained the frequency dependent normalized field amplitude and field enhancement factor through the nanogap with and without substrate as presented in Fig. 3d. The open black squares (the amplitude was multiplied by 10 for comparability) and the open red circles represent the transmission through the nanogap with and without substrate, respectively. The normalization was done separately with the transmission through the reference samples of Si substrate and free space aperture of the equal area. Our experimental results indicates the field enhancement increased by approximately 15 fold at 0.14 THz when Si substrate was removed out from the same nanogap sample. Some irregularities in transmission curve was also observed because of the grating modes when the slit array diffracted the incident radiation into the sample plane. This results can be explained by poor coupling of THz wave among the evanescent modes because of higher refractive index of substrate^29^. The effective refractive index for substrate is given by 
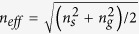
. where n_s_ and n_g_ are refractive index of substrate and gap material, respectively. In our sample, the substrate is Si (n ≈ 3.417) and gap material is Al_2_O_3_ (n ≈ 3.32). Hence, the nanoslit responses to an effectively smaller wavelength *λ*_*eff*_ = *λ*_0_/*n*_*eff*_, where λ_0_ denotes free space wavelength that reduces the transmission and correspondingly it yields a lower field enhancement^22^.

### Nanoslit fabrication methods

**Figure 4:**
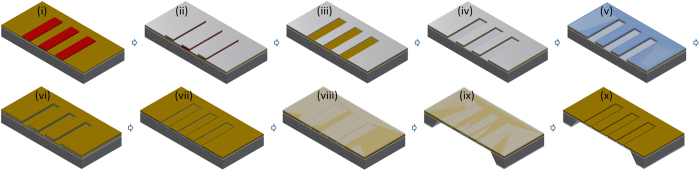
Nanoslit fabrication flow chart. (i) Patterning on metal-coated Si substrate by photolithography, (ii) sacrificial layer (Cr/Al) deposition by e-beam evaporation, (iii) lift-off by acetone and sonication, (iv) ion milling of 1^st^ metal layer, (v) gap width definition via ALD (Al_2_O_3_), (vi) 2^nd^ metal layer deposition, (vii) revealing the ALD nanoslits by chemical etching of the sacrificial layer, (viii) nanogap protection layer (SiO_2_) deposition by PECVD, (ix) back-side bulk Si etching to reveal oxide/nitride membrane, and (x) exposure of the nanoslits by etching the SiO_2_ protection layer and Al_2_O_3_ with BHF.

We fabricated the gold nanoslits on the large area SiN_x_ membrane by using atomic layer lithography and standard microfabrication techniques on a 4” Si wafer. A schematic flow chart of the fabrication process is presented in Fig. 4. We first fabricated the ALD nanogap in the metal on the Si substrate and then used atomic layer lithography and plugged and lifted off the excess metal by etching the sacrificial layer^26^. Then, the back-side bulk Si was etched out, thus resulting in nanoslits on the SiO_2_/Si_3_N_4_ membrane. Finally, Al_2_O_3_ and the SiO_2_ layer were etched out, thereby releasing the air gap gold nanoslits.

The process began with the thermal oxidation of a 4 inch Si wafer (〈100〉, p-type, 10 ohm.cm resistivity) to make 1-μm SiO_2_ as a passivation layer, and this was followed by low pressure chemical vapour deposition (LPCVD) to grow 1-μm low-stress silicon nitride. After the first metal layer (Au: 100 nm) was deposited, patterning on the metal film was completed by photolithography (Fig. 4i), and this was followed by sacrificial layer/etch mask deposition (Fig. 4ii), lift-off (Fig. 4iii), and ion milling (Fig. 4iv). An Al_2_O_3_ layer was deposited (Fig. 4v) by ALD at 250 °C on the sidewall of the Au film. In ALD, Tri-methylaluminium and water vapour were sequentially pulsed through the chamber, with N_2_ purging after each injection, until 19 cycles yielded 5-nm-thick Al_2_O_3_. Subsequently, the second metal layer (Au 95 nm) was deposited on top of the first (Fig. 4vi), and the excess 2^nd^ layer was lifted off (Fig. 4vii) by etching the sacrificial layer. The lift-off of the second layer removed excess metal and opened up the ALD layer in between the first and second layers. 300-nm-thick aluminium was used as both an etch mask for ion milling and a sacrificial layer to lift off the excess 2^nd^ layer on top of it. The Al etchant (1 M KOH, or any strong alkaline solution at room temperature) entered through the intentionally uncovered Au area, etched the Al, and lifted off the excess 2^nd^ layer Au film. The etchant also slightly etched Al_2_O_3_, and ultra-sonication and cleaning in acetone, methanol and deionized (DI) water were performed to remove residues at the nanogap. Further surface polishing of the nanogap was carried out with an almost vertical (85°) Ar ion milling process.

The back-side bulk silicon was selectively etched out (Fig. 4ix) through the pre-defined window by a tetramethylamoniumhydroxide (TMAH 20 wt%) solution at 90 °C, after the deposition of the protective SiO_2_ layer (Fig. 4viii) on the top of the nanogap surface. The SiO_2_ protected the nanogap and supported the etching of Si by preventing the metal-coated Si substrate from forming as an etch shield. The protective layer was later etched out by dipping it in HF solution, which also removed the bottom SiO_2_ layer (Fig. 4x). Dipping the sample in HF also etched the alumina between the metal, thereby exposing the air gap in the metal. To release the fully free-standing nanogap metal membrane for material transportation through the slits, the remaining thin Si_3_N_4_ membrane was also etched out with reactive ion etching (RIE). In most cases, after etching the nitride membrane, the gold membrane became unstable and collapsed. The nanogaps either collapsed or, where the bridge-like structure survived, widened. Thus, we kept the robust nitride membrane to support the metallic nanoslits and increase the stability of the nanogap for the THz experiments.

## Conclusion

A large nonresonant electromagnetic field enhancement was experimentally realized with practically infinite gold nanoslits. The field enhancement was almost 10^4^ in the lower THz frequency regime without any resonance peak, and then it monotonously decreased, following the 1/f curve. The large field enhancement in the nanogap region can be explained by the nanocapacitor model, and is formed as a result of the excitation of SPPs when the THz field is incident on the metal surface. The non-resonance effect was attributed to the practically infinite (length-to-width ratio ~10^5^) and substrate-free nature of the nanoslits. The substrate-free nanoslits were fabricated using plug-and-lift-off and standard Si microfabrication techniques in which the gap width and uniformity were determined by atomic layer lithography. The yield of such an ultra-thin nanoslit membrane depends on the strength of the metal microribbon, with negligible deformities due to temperature and pressure. The developed substrate-free nanoslits may be useful in studies of gap plasmonics, quantum tunnelling, nonlinear optics, and nanophotonics, for which high field enhancement is important.

## Additional Information

**How to cite this article:** Suwal, O. K. *et al*. Nonresonant 10^4^ Terahertz Field Enhancement with 5-nm Slits. *Sci. Rep.* **7**, 45638; doi: 10.1038/srep45638 (2017).

**Publisher's note:** Springer Nature remains neutral with regard to jurisdictional claims in published maps and institutional affiliations.
